# BIDS-formatted resting-state and loudness dependence of auditory evoked potentials (LDAEP) EEG dataset from healthy women

**DOI:** 10.1016/j.dib.2026.112940

**Published:** 2026-06-06

**Authors:** Henrik Normannseth, Stein Andersson, Christoffer Hatlestad-Hall

**Affiliations:** a Section for Clinical and Cognitive Neuroscience, Department of Psychology, University of Oslo, P.O. Box 1094, Blindern 0317 Oslo, Norway; b Department of Psychiatry, Lovisenberg Diaconal Hospital, P.O. Box 4970, Nydalen 0440 Oslo, Norway; c Section of Psychosomatic Medicine, Division of Mental Health and Addiction, Oslo University Hospital, P.O. Box 4956, Nydalen 0424 Oslo, Norway; d Department of Neurology, Oslo University Hospital, P.O. Box 4950, Nydalen 0424 Oslo, Norway

**Keywords:** Electroencephalography, Event-related potentials, Women's health, Hormonal contraceptives, Sex hormones, Depressive symptoms

## Abstract

Here, we describe a publicly available electroencephalography (EEG) dataset recorded from 69 healthy women (aged 19–40). Data were acquired using a 64-channel BioSemi ActiveTwo system (10–10 electrode positions) at a sampling rate of 2048 Hz. The dataset includes two EEG paradigms: (1) a resting-state recording consisting of four minutes with eyes closed followed by four minutes with eyes open (available for all 69 participants), and (2) a loudness dependence of auditory evoked potentials (LDAEP) paradigm comprising 1000 Hz tones at five intensity levels (55–95 dB SPL; available for 54 participants). The dataset also includes demographic variables, hormonal contraceptive (HC) usage information, menstrual cycle data, Beck Depression Inventory-II (BDI-II) scores, and UPPS Impulsive Behavior Scale subscale scores. The data are organized according to the Brain Imaging Data Structure (BIDS) specification and hosted on OpenNeuro. The dataset is suitable for research on resting-state EEG, LDAEP, hormone-related variation in brain electrophysiology, and machine learning applications.

Specifications Table

**Table:** 

Subject	Health Sciences, Medical Sciences & Pharmacology
Specific subject area	Electrophysiology and psychoneuroendocrinology in women's mental health.
Type of data	Raw electroencephalography (.eeg, .vhdr, .vmrk), tabular data and metadata (.tsv, .json).
Data collection	EEG data were recorded using a 64-channel BioSemi ActiveTwo system (10–10 positions) at 2048 Hz. Resting-state: 4 min eyes closed, 4 min eyes open. The LDAEP paradigm presented 1000 Hz tones at five intensities (55–95 dB SPL, 80 trials each) via Etymotic ER-1 earphones, generated using Psychtoolbox-3 in MATLAB. Inclusion: women aged 18–40, normal hearing, not pregnant/breastfeeding. Exclusion: psychiatric/neurological disorders, substance abuse, psychoactive medications. Questionnaires: Beck Depression Inventory-II (depressive symptoms), UPPS Impulsive Behavior Scale (trait impulsivity). Data organized in BIDS format.
Data source location	Institution: Department of Psychology, Faculty of Social Sciences, University of Oslo. City: Oslo. Country: Norway.
Data accessibility	Repository name: OpenNeuro Data identification number: ds007615 Direct URL to data: https://doi.org/10.18112/openneuro.ds007615.v1.0.0
Related research article	H. Normannseth, C. Hatlestad-Hall, T.W. Rygvold, A. Hadzic, S. Andersson, Hormonal contraceptive use is associated with reduced central serotonergic activity indexed by the loudness dependence of auditory evoked potentials, *Frontiers in Human Neuroscience*, 19 (2025) 1647,425. https://doi.org/10.3389/fnhum.2025.1647425

## 1 Value of the Data

• Publicly available loudness dependence of auditory evoked potentials (LDAEP) datasets are scarce. The current dataset provides an opportunity for researchers to develop and validate analysis methods for auditory intensity-dependent paradigms without the need to collect new data.
• The combination of LDAEP data, resting-state electroencephalography (EEG), and detailed hormonal contraceptive (HC) data enables investigation of sex hormone and brain interactions, a topic of growing importance in psychoneuroendocrinology and women's health research.
• The inclusion of Beck Depression Inventory-II (BDI-II) depression scores and UPPS impulsivity subscale scores alongside the electrophysiological data supports analyses relating EEG measures, including LDAEP, to self-reported subclinical mood variation and trait impulsivity.
• The resting-state data, comprising both eyes-closed and eyes-open conditions, offer broad reuse potential for analyses, including spectral power, functional connectivity, graph theory, microstate analyses, and comparisons across resting-state conditions.
• The dataset may be suited for machine learning applications, such as the classification of HC status or prediction of mood and impulsivity scores from electrophysiological features.

## 2 Background

This dataset was compiled in the context of a study examining whether HC use is associated with variation in central serotonergic activity in healthy women, indexed by LDAEP [1]. The data collection was motivated by interest in the relationship between HC use and serotonin-related EEG measures. In addition, the dataset includes resting-state EEG, together with demographic variables, HC use variables, menstrual-cycle-related information, depressive symptom scores, and impulsivity scores. In the original research article [1], the LDAEP was compared between HC users and non-users; HC use was associated with a steeper LDAEP slope, interpreted as reduced central serotonergic activity. The present data article makes the raw EEG and participant-level phenotypic data publicly available [2] and provides a detailed description of the repository contents to support reanalysis of the LDAEP data, resting-state EEG investigations, and secondary analyses of hormonal and phenotypic variables.

## 3 Data Description

The dataset is organized according to the electroencephalography extension of the Brain Imaging Data Structure (BIDS) specification [3]. The general BIDS specification is described in detail at https://bids.neuroimaging.io/ [4]. In the following, '##' (two digits) refers to the sequential subject number (01–69).

### 3.1 Root-level files and directories

The following files and directories are found at the dataset's root level:

• **dataset_description.json:** Metadata file describing the dataset parameters, including the dataset name, authors, and BIDS version.
• **participants.tsv:** Tab-separated text file containing core demographic and grouping variables for each participant (age, sex, education level, and HC user group). A sidecar *participants.json* file describes each variable.
• **phenotype/:** Directory containing additional participant-level data organized into four tab-separated files with sidecar JSON files*: hc_usage.tsv* (HC usage details and menstrual cycle data; 26 variables), *lifestyle.tsv* (medication, nicotine, alcohol, and recreational drug use; 7 variables), *bdi.tsv* (BDI-II total and subscale scores; 4 variables), and *upps.tsv* (UPPS impulsivity subscale scores; 5 variables). The *bdi.tsv* and *upps.tsv* files contain complete data for all 69 participants; no questionnaire scores required imputation. In *hc_usage.tsv*, n/a denotes items not applicable to a given participant.
• **README:** Text file providing an overview of the dataset.
• **CHANGES:** Text file documenting version history.
• **sub-##/:** Subject directories containing raw EEG data.

### 3.2 Subject directories and raw data

Each subject directory (*sub-##/*) contains a *sub-##_scans.tsv* file and an *eeg/* subdirectory with the raw EEG data files. The *sub-##_scans.tsv* file contains information on the time of day when the recording was conducted. The dates have been altered to maintain anonymity. Up to three tasks are included per participant: eyes-closed resting-state (label: *task-restec*), eyes-open resting-state (label: *task-resteo*), and the LDAEP paradigm (label: *task-ldaep*). The resting-state tasks are available for all 69 participants, while the LDAEP data are available for 54 participants; data for the remaining 15 participants were excluded due to auditory stimulation equipment issues identified after data collection (see Experimental protocol below).

Data for each task are stored in BrainVision format, comprising three files: a data file (*.eeg), a header file (*.vhdr), and a marker file (*.vmrk). Each task is additionally accompanied by a sidecar metadata file (*.json) specifying recording parameters (sampling frequency, reference scheme, hardware filters, task description), a tab-separated channels file (*_channels.tsv) listing all data channels with their type, unit, and status (default; no status assessment has been done), and an events file (*_events.tsv with accompanying *_events.json) documenting stimulus and experimental markers. The events files contain start and end markers for the resting-state data, and stimulus type and onset latency for the LDAEP data. The five LDAEP stimulus intensities are labeled ldaep_55, ldaep_65, ldaep_75, ldaep_85, and ldaep_95 in the trial_type column of each *_events.tsv, where the numeric suffix denotes the nominal intensity in dB SPL; the corresponding numerical trigger values (51 to 55) are preserved in the value column and in the original BrainVision marker file (*.vmrk).

### 3.3 Participant and phenotype data

Core demographic and grouping variables are provided in the root-level *participants.tsv* file, which contains five variables: participant identifier, age, sex (all female), HC user group (current user, past user, or never-user), and education level. Variable descriptions are found in the *participants.json* sidecar.

Additional participant-level data are organized in the *phenotype/* subdirectory according to BIDS conventions [3]. The directory contains four files, each accompanied by a JSON sidecar with variable descriptions and coding schemes:

• **hc_usage.tsv:** Detailed HC and menstrual cycle variables (26 columns). The main variable groups are:
  • Current HC use: contraceptive type (combined vs. progestin-only), specific progestin type, oral vs. non-oral administration, duration of use, and reasons for use;
  • Self-reported effects of current use: mood changes and side effects;
  • Prior HC use history: previous use, types, and duration;
  • Medical and reproductive history: hormonal treatment for medical conditions and pregnancy history;
  • Menstrual cycle: phase at the time of recording.
• **lifestyle.tsv:** Lifestyle and substance use variables (7 columns), including medication use, daily nicotine use and type (snus or e-cigarette), weekly alcohol consumption, and recreational drug use and frequency.
• **bdi.tsv:** BDI-II scores (4 columns): total score, cognitive-affective subscale score, and somatic subscale score. Subscale scores follow the female-specific factor structure described in Dozois et al. [5].
• **upps.tsv:** UPPS Impulsive Behavior Scale subscale scores (5 columns): lack of perseverance, urgency, lack of premeditation, and sensation seeking [6].

## 4 Experimental Design, Materials and Methods

The dataset was generated within the study reported in Normannseth et al. [1]. The present section summarizes methodological details relevant to reuse of the EEG and participant-level data.

### 4.1 Participants

The dataset comprises EEG data from 69 healthy women (mean age = 25.3 years; range = 19–40; SD = 4.2). Of these, 37 were current HC users (16 combined HC, 21 progestin-only) and 32 were non-users (21 past users and 11 never-users). Participants were recruited through flyers posted at the University of Oslo, Oslo, Norway. Inclusion criteria were age between 18 and 40 years, normal hearing, and not currently pregnant or breastfeeding. Exclusion criteria included previous or current psychiatric or neurological disorders, substance abuse, or use of psychoactive medications. Table 1 presents descriptive statistics for key participant-level variables, organized by HC user group.

**Table 1:** Participant characteristics by group. Note. Continuous variables are presented as mean ± SD (range). Categorical variables are presented as n (%). HC = hormonal contraceptive; BDI-II = Beck Depression Inventory-II. Education levels correspond to the Norwegian educational system. '—' indicates variable not applicable to the group.

Variable	Current HC users (n = 37)	Past users (n = 21)	Never-users (n = 11)	Total (N = 69)
Age (years)	25.0 ± 3.8 (19–40)	26.9 ± 4.8 (20–40)	22.9 ± 2.8 (20–29)	25.3 ± 4.2 (19–40)
*Education*				
Upper secondary, vocational	0 (0.0%)	0 (0.0%)	1 (9.1%)	1 (1.4%)
Upper secondary, academic	18 (48.6%)	5 (23.8%)	6 (54.5%)	29 (42.0%)
University/university college	19 (51.4%)	16 (76.2%)	4 (36.4%)	39 (56.5%)
*HC subtype*				
Progestin-only	21 (56.8%)	—	—	—
Combined (progestin + estrogen)	16 (43.2%)	—	—	—
BDI-II total	9.7 ± 8.9 (0–36)	6.5 ± 5.1 (0–21)	6.0 ± 5.4 (0–19)	8.1 ± 7.6 (0–36)

### 4.2 Questionnaires

Participants completed questionnaires assessing HC usage history (current and prior use, type, duration, experienced side effects), mood effects attributed to current or prior HC use, and general health and lifestyle factors. HC-related variables were collected using study-specific questionnaire items developed for the original study. Depressive symptoms over the preceding two weeks were quantified using the BDI-II [7], administered in its authorized Norwegian edition; the psychometric properties of the Norwegian version in adults have been documented [8]. BDI-II scores were decomposed into cognitive-affective and somatic subscales following the female-specific factor structure described by Dozois et al. [5]. Trait impulsivity was assessed using the UPPS Impulsive Behavior Scale [6], administered in a Norwegian translation prepared, with permission from the original authors, by Løvstad, Rike, Solbakk, Schanke, and Schanche. The UPPS yielded four subscale scores: lack of perseverance, urgency, lack of premeditation, and sensation seeking. All scores were computed as item sums.

### 4.3 Experimental protocol

Before EEG recording, participants underwent audiometric screening to confirm normal hearing, using a Micromate 304 screening audiometer (Madsen Electronics; EN 60,645–1 type 4). Recordings took place in a sound-attenuated room under low ambient lighting. The EEG session began with a resting-state recording consisting of two consecutive blocks. In the first block (four minutes), participants were instructed to sit with their eyes closed. A brief auditory tone then indicated the transition to the second block (four minutes), during which participants were instructed to open their eyes and fixate on a red dot displayed centrally on a 24-inch LCD screen (BenQ, model ID: XL2420-B). Participants were asked to minimize facial movements throughout the recording.

Following the resting-state recording, participants immediately completed the LDAEP paradigm, in which binaural 1000 Hz pure tones (30 ms duration) were presented at five intensity levels (55, 65, 75, 85, and 95 dB SPL). Each level was repeated 80 times in pseudorandomized order, with inter-stimulus intervals drawn from 1200 to 1800 ms. Tones were generated in MATLAB R2015a (MathWorks, Natick, MA, USA) using Psychtoolbox-3 [9], routed through an Alto AMX-80 mixing console (inMusic Brands, Cumberland, RI, USA), and delivered via Etymotic ER-1 insert earphones (Etymotic Research, Inc.). During this block, participants continued to fixate on the central red dot. A technical issue with the auditory stimulation equipment was identified after data collection for 15 of the 69 participants: the delivered sound pressure levels did not scale across the five nominal intensity steps as specified by the paradigm. Their LDAEP data are therefore omitted, while resting-state recordings and questionnaire data are retained.

### 4.4 EEG acquisition

EEG data were recorded using a 64-channel (Ag-AgCl active electrodes) BioSemi ActiveTwo system (BioSemi B.V., Amsterdam, The Netherlands). Electrodes were positioned according to the extended 10–20 system (10–10) [10]. Data were acquired at a sampling rate of 2048 Hz. The ActiveTwo is DC-coupled and applies a hardware antialiasing filter during acquisition. Four additional electrodes placed around the eyes (two lateral, two vertical) were used to monitor ocular activity for subsequent artifact rejection. Electrode offsets were monitored in real time using BioSemi ActiView (v7.07; BioSemi B.V., Amsterdam, The Netherlands) and kept below ±20 mV before each recording. During acquisition, the system used a Common Mode Sense (CMS) and Driven Right Leg (DRL) feedback loop in place of conventional ground and reference electrodes. Channel-level metadata are provided in each *_channels.tsv; the status field is set to the BIDS default, as no per-channel quality assessment was performed.

The released data are continuous EEG recordings and require preprocessing appropriate to the intended analysis. At minimum, users should address slow drifts and DC offset, for example by applying a suitable high-pass filter, and should detect, reject, or correct ocular and movement-related artifacts using the dedicated ocular channels. The released data are average-referenced and may be re-referenced as required. For LDAEP analyses, additional steps such as low-pass filtering, segmentation into epochs around the stimulus events (trial labels *ldaep_55* to *ldaep_95*), and baseline correction are required. These and other event annotations are available directly in the released *_events.tsv, *_events.json, and *.vmrk files.

## Limitations

The dataset has several limitations. First, the sample is modest in size (N = 69) and homogeneous: it consists exclusively of healthy women aged 19 to 40 recruited at a single Norwegian university, which limits generalizability to other populations, age groups, and geographic or cultural settings. Second, LDAEP data are unavailable for 15 of the 69 participants due to a technical issue with the auditory stimulation equipment (see Experimental protocol); their resting-state and questionnaire data are retained. As a result, analyses linking LDAEP to the phenotypic variables are limited to the 54 participants with usable LDAEP data. Third, certain phenotype variables rely on self-report, including depressive symptoms, HC usage details, and menstrual cycle phase, which may reduce measurement precision. Lastly, no salivary or serum hormone measurements were collected, so hormonal status is characterized by self-reported HC use and cycle information rather than by direct hormone assays.

## Ethics Statement

The data collection was conducted in accordance with the Declaration of Helsinki, and written informed consent was obtained from all participants. The procedures were approved by the Regional Ethics Committee of South-Eastern Norway (reference number: 657256, approved 15 September 2023). All participants received compensation equivalent to approximately 30 USD for their participation.

## CRediT Author Statement

**Henrik Normannseth:** Data curation, Investigation, Methodology, Software, Writing - original draft; **Stein Andersson:** Conceptualization, Funding acquisition, Investigation, Methodology, Project administration, Supervision, Writing - review & editing; **Christoffer Hatlestad-Hall:** Data curation, Methodology, Software, Supervision, Writing - review & editing.

## Data Availability

OpenNeuroLDAEP and resting-state EEG in healthy women (Original data)

## Appendix Supplementary materials

Image, application 1
